# Temperature, energy metabolism, and adaptive divergence in two oyster subspecies

**DOI:** 10.1002/ece3.3085

**Published:** 2017-06-30

**Authors:** Ao Li, Li Li, Kai Song, Wei Wang, Guofan Zhang

**Affiliations:** ^1^ Key Laboratory of Experimental Marine Biology Institute of Oceanology Chinese Academy of Sciences Qingdao Shandong China; ^2^ University of Chinese Academy of Sciences Beijing China; ^3^ Laboratory for Marine Fisheries and Aquaculture Qingdao National Laboratory for Marine Science and Technology Qingdao Shandong China; ^4^ National & Local Joint Engineering Key Laboratory of Ecological Mariculture Institute of Oceanology Chinese Academy of Sciences Qingdao Shandong China; ^5^ Laboratory for Marine Biology and Biotechnology Qingdao National Laboratory for Marine Science and Technology Qingdao Shandong China

**Keywords:** *Crassostrea*, gene expression, genetic variation, phenotypic plasticity, physiology, tradeoffs

## Abstract

Comparisons of related species that have diverse spatial distributions provide an efficient way to investigate adaptive evolution in face of increasing global warming. The oyster subjected to high environmental selections is a model species as sessile marine invertebrate. This study aimed to detect the adaptive divergence of energy metabolism in two oyster subspecies from the genus *Crassostrea*—*C. gigas gigas* and *C. gigas angulata*—which are broadly distributed along the northern and southern coasts of China, respectively. We examined the effects of acute thermal stress on energy metabolism in two oyster subspecies after being common gardened for one generation in identical conditions. Thermal responses were assessed by incorporating physiological, molecular, and genomic approaches. Southern oysters exhibited higher fluctuations in metabolic rate, activities of key energetic enzymes, and levels of thermally induced gene expression than northern oysters. For genes involved in energy metabolism, the former displayed higher basal levels of gene expression and a more pronounced downregulation of thermally induced expression, while the later exhibited lower basal levels and a less pronounced downregulation of gene expression. Contrary expression pattern was observed in oxidative stress gene. Besides, energy metabolic tradeoffs were detected in both subspecies. Furthermore, the genetic divergence of a nonsynonymous SNP (*SOD‐132*) and five synonymous SNPs in other genes was identified and validated in these two subspecies, which possibly affects downstream functions and explains the aforementioned phenotypic variations. Our study demonstrates that differentiations in energy metabolism underlie the plasticity of adaptive divergence in two oyster subspecies and suggest *C. gigas angulata* with moderate phenotypic plasticity has higher adaptive potential to cope with exacerbated global warming.

## 1 INTRODUCTION

Examination and prediction for adaptive potential of marine organisms is a vital evolutionary mission in the context of global climate change. Intertidal species, which recruit physiological or evolutionary responses to persistent oceanographic variations in temperature, nutrients, and other abiotic/biotic parameters, are particularly deserved to explore these issues (Sanford & Kelly, 2011). Of the varying marine environmental stressors, temperature is considered to be the central selective factor because of its profound effect on the organisms’ biochemistry and physiology. Climate change further exacerbates the stresses on the intertidal organism, particularly due to the forecasted  global warming of 3°C–5°C by 2,300 (Dineshram et al., 2016; Somero, 2012; Stocker et al., 2014). The increased temperature of the upper layers of the ocean may have inevitable impacts on marine ectotherms at the cellular, individual, and population levels (Bernhardt & Leslie, 2013; Schulte, 2015). Thus, it is urgent to assess the adaptive capacity of intertidal invertebrates to cope with large environmental fluctuations on the short‐ or long‐term timescale.

Changes in environmental conditions are often correlated with shifts of species’ distribution patterns from biogeographic to local scales (Somero, 2012). And geological range of a given species reflects evolutionary responses of populations in genomic, transcriptomic, and physiological functions from two forms: local adaptation and phenotypic plasticity. These two evolutionary responses may evolve together as adaptive phenotypic plasticity when adjustments in metabolism and physiology of a genotype are adapted to local environments (Bozinovic, Calosi, & Spicer, 2011; Yampolsky, Schaer, & Ebert, 2014). Oysters are intertidal bivalve mollusks and an ideal model for studies of adaptive evolution due to its sessile habit, high degree of genomic polymorphism, and phenotypic plasticity (Guo, He, Zhang, Lelong, & Jouaux, 2015; Zhang et al., 2012). Of the genus *Crassostrea*,* Crassostrea gigas gigas* (Thunberg 1793) and *C. gigas angulata* (Lamarck 1819) inhabit the northern and southern intertidal areas of the Chinese coastline, respectively, and occupy distinct ecological niches (Wang, Qian, Liu, Zhang, & Guo, 2010; Wang, Zhang, Liu, & Guo, 2008). They diverged approximately 2.7 Mya and have been identified as two subspecies (Ren, Liu, Jiang, Guo, & Liu, 2010).

Adaptive variations among closely related species exert an important influence on many aspects of animal ecology and evolution, and appear to be both consistent over time and pervasive among marine species (Reale, Reader, Sol, McDougall, & Dingemanse, 2007; Reale et al., 2010). Phenotypic plasticity is the most prominent factor in the maintenance of these differences across spatial and temporal dimensions (Bateson, 2015). Previous studies have clarified the functional linkage between energetics and consistent differences, where energy metabolism is believed to exhibit adaptive features under different environmental and developmental conditions (Careau, Thomas, Humphries, & Reale, 2008). Energy homeostasis is a fundamental requirement for cellular function and survival, as well as for stress adaptation. Alternatively, in the absence of homeostasis, energy metabolism would be strongly affected by environmental stressors, which require organisms to supply additional energy costs; destroy the balance of energy acquisition, conversion, and conservation; and induce tradeoffs in energy allocation (Killen, Marras, Metcalfe, McKenzie, & Domenici, 2013; Sokolova, Frederich, Bagwe, Lannig, & Sukhotin, 2012). This regulatory framework is consistent with the concept of energy‐limiting stress tolerance and could be used to investigate physiological and energetic consequences in the face of different ecological stressors on both interspecific or intraspecific levels (Sokolova et al., 2012). This hypothesis proposes that environmental factors can affect energy balance and windows of tolerance, resulting in energetic tradeoffs and shifting from aerobic to anaerobic metabolism. In particular, aerobic thresholds are under strong environmental selection and shape species‐specific responses to climate change (Killen et al., 2013; Schulte, 2015).

Mechanistic studies of the geographic patterns of a given species in response to environmental gradients can be best achieved by integrating genomic and physiological information (Bozinovic et al., 2011; Doney, 2010). In fact, it is difficult to conclude whether observed patterns in population structure are the result of adaptive divergence (natural selection) or are a result of random processes such as genetic drift by observing genetic differences or physiological variations in isolation (Burford, Scarpa, Cook, & Hare, 2014). Genetic differentiation in energy metabolism has been considerably investigated in marine fishes (Limborg et al., 2012) and bivalves (Ni, Li, Kong, & Zheng, 2012; Zhan et al., 2009). In addition, adaptive variations in physiological responses have also been investigated in many molluskan species, such as marine snails (Sokolova & Pörtner, 2001), blue mussels (Tomanek & Zuzow, 2010), an intertidal limpet (Han, Zhang, Marshall, Ke, & Dong, 2013), and oysters of the genus *Crassostrea* (Lannig, Eilers, Pörtner, Sokolova, & Bock, 2010; Sussarellu et al., 2012). Moreover, gene expression profiles help to link genotype to phenotype and play a central role in cellular adaptations to environmental change. Differential selection between environments could result in the coevolution of correlated transcriptomic responses and quantitative trait variation (Kenkel & Matz, 2016; Larsen, Schulte, & Nielsen, 2011). Metabolic genes have been recognized as molecular markers of adaptive divergence in marine organisms, especially in studies concerning the influence of thermal fluctuations in face of climate change (Kenkel, Meyer, & Matz, 2013; Whitehead & Crawford, 2006).

In this study, we collected individuals of *C. gigas gigas* and *C. gigas angulata* from their native habitats and common‐gardened them in the identical conditions for one generation to avoid potential interactions between environmental and genetic effects on phenotype (Reale et al., 2007; Sanford & Kelly, 2011). A complete picture of the adaptive divergence of these two subspecies was assessed from the perspective of energy metabolism by observing the effects of thermal stress on metabolic rate, the activities of key metabolic enzymes, the expression levels of genes involved in energy metabolism, and the genomic variations of SNPs in these genes and its subsequent functional analyses. This work, from genotype to phenotype, reveals the underlying mechanisms of adaptive divergence from the view of energy metabolism in two oyster subspecies and assess their evolutionary potential of phenotypic plasticity, which not only enhanced our ability to predict biological responses of intertidal invertebrates to future warming, but assist the development of resource conservation to mitigate the impacts of climate change.

## 2 MATERIALS AND METHODS

### 2.1 Common garden experiments

**Figure 1:**
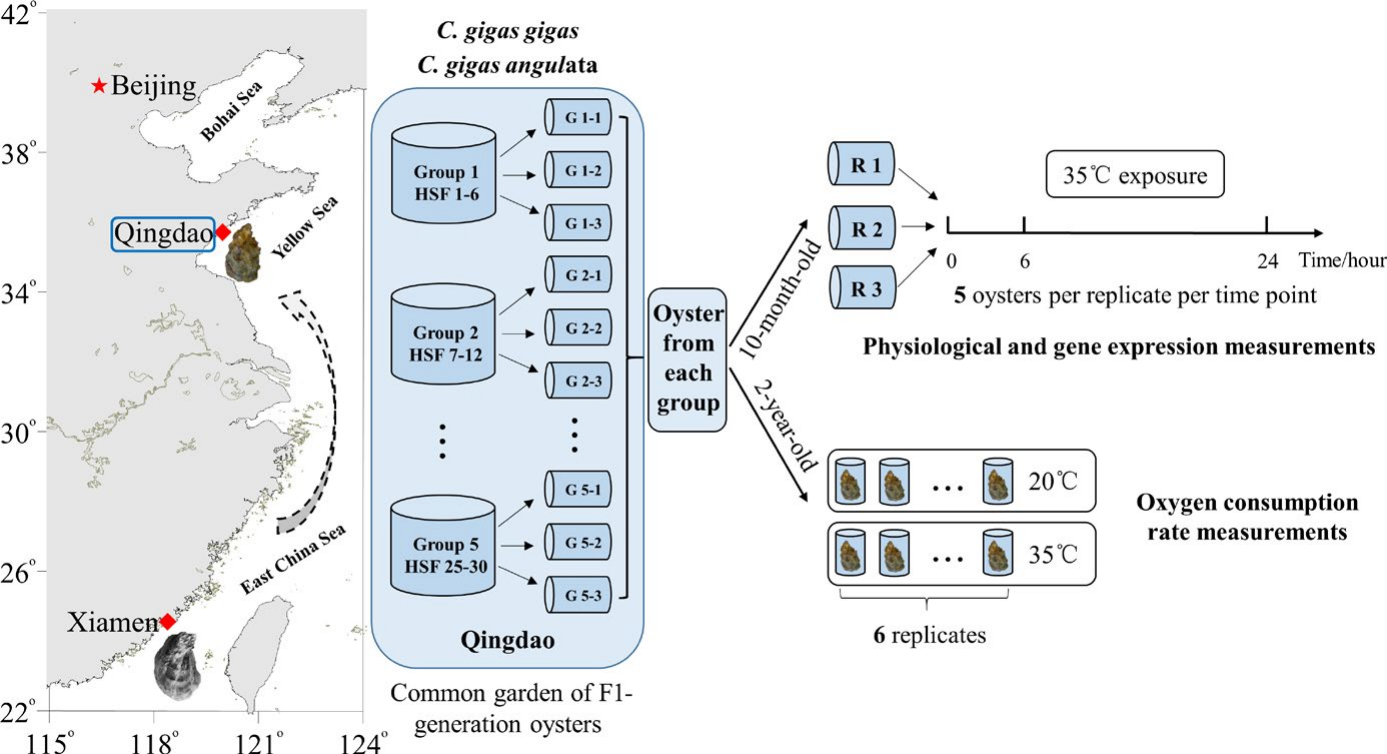
Common garden experiments and experimental designs. For each of two oyster subspecies, *Crassostrea gigas gigas* and *C. gigas angulata*, wild parental oysters were transported to Qingdao for acclimation. Eggs of 30 sexual maturity female oysters were mixed and divided into 30 beakers, and then fertilized with each of 30 male oysters. At the D‐shaped stage, oyster larvae from six half‐sib families (HSF) were combined into one group as biological replicate. In total, five groups (G) with three replicates of each were cultured in Qingdao. Ten‐month‐old oysters were used for physiological and gene expression measurements. Oysters from each group (biological replicates) were merged into three replicates (R) before experiments. Animals were exposed into 35°C seawater. Gills and mantles of five oysters from each replicates were collected at each time points of 0, 6, and 24 hr, which used to determine gene expression and physiological parameters, respectively. Six two‐year‐old oysters of each subspecies were used to measure oxygen consumption rate at 20°C and 35°C

Wild parental specimens of two oyster subspecies, *C. gigas gigas* and *C. gigas angulata*, were collected from the intertidal area of the Yellow Sea (35°44′N, Qingdao, Shandong province, China) and the East China Sea (24°33′N, Xiamen, Fujian province, China), respectively, during May 2014. Collected oysters were transported alive and cultured in the sea in Qingdao before spawning. Mature oysters were inbred within subspecies. In detail, mixed eggs from 30 female oysters were divided into 30 parts and then crossed individually with the gametes of 30 male oysters. Larvae from six males were combined into one group and then divided into three repeats at the D‐shaped stage (Figure 1). The rearing of larvae and spat was performed using standard practices (Guo, Li, Wang, & Kong, 2012). The spat were put into cages at a density of approximately 100 individuals per layer after shell height had reached 3 cm. Cages were changed four times each year, and the density decreased as the sizes of the oysters increased. In addition, air temperature of the latest 2 years from 2015 and 2016 was recorded from weather stations at Qingdao and Xiamen, as it exerts direct effects on the intertidal organisms.

Oysters were cleaned to remove epifauna and acclimated in aquaria for 15 days with aerated and sand‐filtered seawater prior to experimentation. Commercial spirulina powder was added as a food source, and seawater was changed daily. No mortality was detected during laboratory acclimation.

### 2.2 Respiration rate measurement

Adult 2‐year‐old oysters of similar size (*n* = 6, shell height: 86.66 ± 2.31 mm (*C. gigas gigas*); 77.81 ± 5.14 mm (*C. gigas angulata*); *p *=* *.1333) were cleaned by wiping with 50% alcohol to avoid the influence of epiphytes prior to experiments. A water bath was used to regulate temperature at 20°C to simulate ambient conditions and at 35°C to simulate acute heat stress. Individual oysters were placed in 1.2‐L acrylic chambers filled with sand‐filtered, air‐saturated seawater at the desired temperature. Seawater was circulated slowly using a rotating magnetic stir bar beneath the experimental chamber. The needle‐type fiber‐optic oxygen microsensor (oxygen optode) and a temperature probe (PreSens, Regensburg, Germany) were glued into two small holes in the lid. An oxygen transmitter (Microx 4; PreSens) was connected to these two probes and recorded temperature and oxygen concentration every 3 s for 1 hr after a 15‐min period of acclimation to the experimental conditions for each oyster. This was established as the necessary time for obtaining stable measurements of oxygen concentration via preliminary experiments. Oxygen microsensors were calibrated to the corresponding temperature and salinity conditions prior to each trial according to manufacturer's instructions. The slope of the decrease in oxygen concentration was calculated as the respiration rate (mg·mL^−1^·hr^−1^). In addition, we investigated the influence of shell height on respiration rate.

### 2.3 Acute heat stress

Juvenile oysters (10 months old) of similar size (shell height: 33.22 ± 0.41 mm (*C. gigas gigas*,* n* = 150), 32.86 ± 0.55 mm (*C. gigas angulata*,* n* = 99), *p *=* *.599) were randomly selected and divided into three groups for each subspecies. The six groups of oysters were then exposed to acute heat stress (35°C) for 24 hr. Seawater (aerated and sand‐filtered) was not changed, and the oysters were not fed during this period. Tissues for physiological and molecular measurements were sampled at 0, 6, and 24 hr. The gills (molecular) and mantles (physiological) of five oysters per time point of each group were immediately dissected and frozen in liquid nitrogen, and then stored at −80°C for subsequent analyses.

### 2.4 Physiological parameters

For the determination of metabolic products and enzymatic activities, three pools of samples were prepared by pooling ~15 mg frozen mantle of five individuals at each sample time of each subspecies. Mixed tissue was added to an excess volume (9×) of precooled saline and ground using a homogenizer on ice. Precipitates were removed by centrifuging for 10 min at 2500 rpm and 4°C. Supernatants were diluted ninefold using saline and stored at −80°C.

The content of total protein (Total protein quantitative assay kit) and malondialdehyde (Malondialdehyde (MDA) assay kit (TBA method)), and the enzymatic activities of pyruvate kinase (Pyruvate kinase (PK) assay kit) and total ATPase (ATPase assay kit) was measured using the corresponding kits purchased from Nanjing Jiancheng Bioengineering Institute (Nanjing, China), and total superoxide dismutase (SOD) was determined by Total Superoxide Dismutase Assay Kit with NBT (Beyotime, Shanghai, China). Assays performed following the corresponding manufacturer's protocols (Buege & Aust, 1978; Hopkirk & Bloxham, 1980; Sun, Oberley, & Li, 1988), and absorbance values were determined using a Varioskan Flash Multimode Reader (Thermo Fisher Scientific, Waltham, MA, USA).

### 2.5 Expression level of thermal responsive genes

Total RNA was extracted by the RNAprep Pure Tissue Kit (Tiangen, Beijing, China) using approximately 20 mg of frozen gill tissue according to the manufacturer's instructions. The supernatant was treated with DNase I during the extraction. RNA quality was assessed via 1.2% gel electrophoresis. RNA concentrations were measured at 260 nm using a Nanodrop 2000 spectrophotometer. After this step, equal amounts (1 μg) of RNA from five oysters were pooled together at each sample time of each subspecies to alleviate bias if one sample with exceptionally high expression of a given gene. Reverse transcription was carried out using PrimeScript RT reagent Kit (TaKaRa Bio, Shiga, Japan) on 1 μg of total RNA following the manufacturer's instructions. Synthesized cDNA was diluted 20‐fold for the determination of expression.

For this study, 10 genes (eight for energy metabolic pathways and two for the stress response: *PFK*,* HXK2*,* IDH*α, *ACSF3*,* SIRT5*,* ATP*α, *VATPA*,* G3PD*,* HIF1N*, and *SOD*) were screened for mRNA expression using real‐time PCR. The gene of *elongation factor 1 alpha* (*EF1*α) was analyzed as an internal control based on its low expressional variability during acute heat stress (Li, unpublished). The specific primers used here are indicated in Table S1. PCR efficiencies were determined using the slope of standard curves for each primer pair from serial dilutions of cDNA and calculated following the formula: *E *=* *10^(−1/slope)^−1 (Stahlberg, Aman, Ridell, Mostad, & Kubista, 2003). Based on the high conservation of most genes between *C. gigas gigas* and *C. gigas angulata* (unpublished, Qi et al.) and high amplification efficiency (close to 1) (Table S1), we used the same primes for both subspecies.

Quantitative real‐time PCR was performed to measure the expression of candidate genes and carried out in duplicate in a final volume of 20 μL using the ABI7500 Fast Real‐Time Detection System (Applied Biosystems, Foster City, USA), containing 3 μL diluted cDNA, 10 μL SYBR Green 2X Supermix (TaKaRa), 5.8 μL DEPC H_2_O, 0.4 μL of each primer pair, and ROX Dye II. Runs started with a 30‐s activation of DNA polymerase at 95°C, followed by 40 cycles of 5 s at 95°C and 30 s at 60°C. The melt curve program was conducted as follows: 15 s at 95°C, 1 min at 60°C, 30 s at 95°C, and 15 s at 60°C. The abundance of relative basal (0 hr) and induced transcripts (6 hr and 24 hr) was determined using the Livak 2^−△△CT^ method (Livak & Schmittgen, 2001), while the mRNA levels of *C. gigas gigas* were used as control to compare basal gene expression between two subspecies.

### 2.6 Validation of genetic variation of candidate genes

We selected 22 candidate genes (including 11 genes used in the gene expression analysis) that are primarily involved in energy metabolism and screened single nucleotide polymorphisms (SNPs) in the exons of each gene with different genotype frequencies between *C. gigas gigas* and *C. gigas angulata* (Table S2), using resequencing data from wild animals of two oyster subspecies collected in 2013 and the expression levels of thermally responsive genes (Li, unpublished). Independent wild oysters (parental animals of these two subspecies used for common garden experiments collected in 2014, *n* = 100) were used to validate the genotype frequency of candidate genes. The genomic DNA of 100 oysters from each subspecies was isolated from gill tissue using the TIANamp Marine Animals DNA Kit (Tiangen, Beijing, China). The quality and quantity of DNA were determined by agarose gel (1%) electrophoresis and UV spectrometry on a NanoDrop 2000 device, respectively. An improved small‐amplicon high‐resolution melting (HRM) analysis was adopted (Wang et al., 2015).

#### 2.6.1 Primer design and amplification of candidate SNPs

SNPs with no other predicted SNPs in the neighboring 30‐bp regions were selected for PCR amplification. The melting temperature (Tm) of the primers was set between 45°C and 55°C, and amplicon lengths were limited to 40–100 bp to minimize the presence of unpredicted SNPs in the amplified region. The PCR mixture was covered by 15 μL of mineral oil and consisted of 5–10 ng of genomic DNA, 0.5 μL (100 pmol/L) each of the forward and reverse primers, and 5 μL of PCR MIX. The PCR thermal conditions were as follows: an initial denaturation step at 95°C for 3 min; followed by 45 three‐step PCR cycles at 95°C for 30 s, Tm for 30 s, and 72°C for 30 s; and a final extension at 72°C for 10 min. All PCR products were prescreened via 10% polyacrylamide gel electrophoresis (PAGE) to check amplification quality. Primer pairs that produced a single clear band on the gel were selected for subsequent HRM analyses.

#### 2.6.2 SNP validation by HRM analysis

Fluorescent melting curves of PCR amplicon duplexes were analyzed using a Light Scanner 96 device. Two unblocked, double‐stranded oligonucleotides were used as high‐ and low‐temperature internal controls in the experiment to calibrate the temperature variation between reactions (Gundry et al., 2008). The duplex controls consisted of the following sequences and their complements: GCGGTCAGTCGGCCTAGCGGTAGCCAGCTGCGGCACTGCGTGACGCTCAG (high‐temperature sequence), ATCGTGATTTCTATAGTTATCTAAGT AGTTGGCATTAATAATTTCATTTT (low‐temperature sequence). Specifically, 1 μL (100 pmol) of the internal controls and 1 μL of LC‐green were added to the amplification products, and denaturation was performed at 95°C for 10 min using a thermal cycler prior to HRM analysis. Melting curve data were collected using continuous fluorescence acquisition at 55°C–98°C, at a thermal transition rate of 0.1°C/s. Genotypes were identified by the melting temperatures indicated by peaks on the derived plots using the Light Scanner 96 software. The Sequence Manipulation Suite (SMS, http://www.bio-soft.net/sms/index.html) was used to DNA sequence translation. And SWISS‐MODEL (https://www.swissmodel.expasy.org/) was used to predict the three‐dimensional structure of proteins.

### 2.7 Data analyses and statistics

All statistical analyses were carried out using R software (R Development Core Team 2013). Data were checked for normality using a Shapiro–Wilk test and for the homogeneity of variances using a Bartlett test. The comparison of respiration rate and growth parameters between *C. gigas gigas* and *C. gigas angulata* was tested with the function *aov* if the data followed a normal distribution and homoscedasticity of variances. Otherwise, a nonparametric Kruskal–Wallis test was performed. These methods of analyses were also used to compare levels of heat‐induced gene expression between these two subspecies at both sampling times. The simple linear regression between respiration rate and shell height was carried out with the function *lm*. The basal expression levels of *C. gigas angulata* relevant to *C. gigas gigas* were analyzed using a two‐tailed *t* test. To analyze physiological parameters, linear‐mixed models were used, with the fixed factors “species” accounting for *C. gigas gigas* and *C. gigas angulata*, “time” for sampling time, and their interaction. When significant differences owing to fixed factors were observed, following the false discovery rate correlation method of Bonferroni, a post hoc Tukey's honestly significant difference (HSD) test was performed to evaluate each pairwise comparison. Pearson's chi‐square test was used to determine differences in genotype frequency between *C. gigas gigas* and *C. gigas angulata*. Data are presented as means ± SEM.

## 3 RESULTS

### 3.1 Air temperature

There are obvious differences in air temperature (>8°C) between two native habitats, especially in the winter, during 2015 and 2016. The average annual high air temperature was 24.43°C in Xiamen and 15.48°C in Qingdao (Figure S1), and the average annual low air temperature was 18.04°C in Xiamen and 9.62°C in Qingdao (Figure S1).

### 3.2 Respiration rate

**Figure 2:**
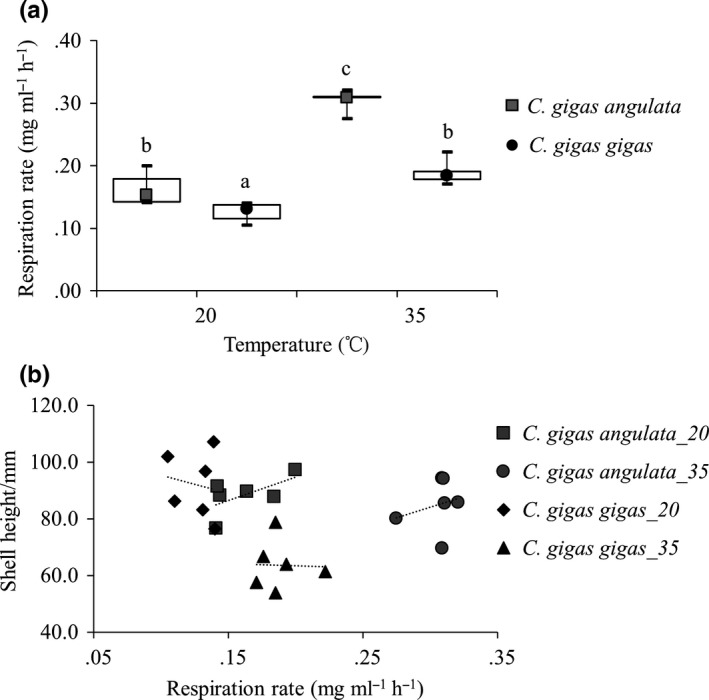
Effects of temperature on the respiration rates of *Crassostrea gigas gigas* and *C. gigas angulata* (a), and the correlations between respiration rate and individual shell height (b) (*n* = 6). Ambient temperature = 20°C and heat stress = 35°C. Letters indicate significance differences between subspecies (*p* < .05, ANOVA)

Significant differences in the respiration rates of these two subspecies were observed, where *C. gigas angulata* exhibited a higher metabolic rate when exposed to both ambient (20°C, *C. gigas gigas*: 0.13 ± 0.0062 mg·mL^−1^·h^−1^, *C. gigas angulata*: 0.16 ± 0.0102 mg·mL^−1^·h^−1^; *p *<* *.05) and high temperatures (35°C, *C. gigas gigas*: 0.19 ± 0.0074 mg·mL^−1^·h^−1^, *C. gigas angulata*: 0.31 ± 0.0064 mg·mL^−1^·h^−1^; *p *<* *.001). In addition, high respiration rates were detected during exposure to high temperature in both subspecies, where *C. gigas angulata* exhibited higher flexibility (1.49‐fold change in *C. gigas gigas* and 1.99‐fold change in *C. gigas angulata*;* p *<* *.001) (Figure 2a). There was no correlation between respiration rate and shell height in both subspecies (*p *>* *.05) (Figure 2b).

### 3.3 Physiological parameters

**Figure 3:**
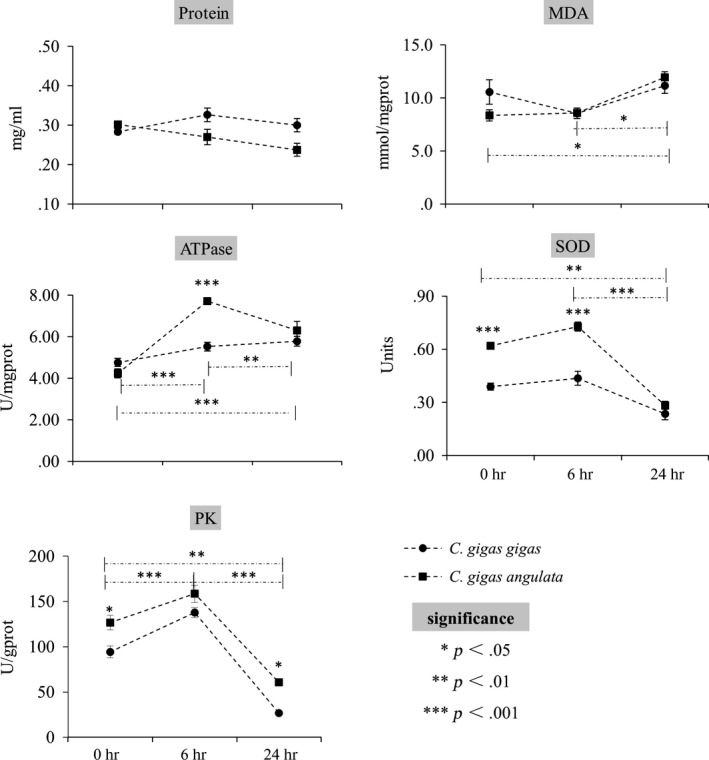
Physiological parameters of *Crassostrea gigas gigas* and *C. gigas angulata* during acute heat stress. Heat stress=35°C (*n* = 15). Asterisks indicate the significance of linear‐mixed modes (*p* < .05). Asterisks with a dashed line above indicate significant differences between sampling times in both subspecies (between three sampling times), while a line below represents differences only in *C. gigas angulata*. Significant differences between two subspecies are indicated with individual asterisks. Vertical bars represent standard errors. MDA, malondialdehyde; SOD, superoxide dismutase; PK, pyruvate kinase

Although no significant differences in total protein and MDA concentrations were detected between *C. gigas gigas* and *C. gigas angulata*, there were significant effects of time on MDA concentration in *C. gigas angulata*, which displayed a tendency to accumulate during exposure to high temperature (*p *<* *.05). In addition, there were obvious disparities in the activities of enzymes between these two subspecies (Figure 3). Specifically, the enzyme activities of total ATPase, SOD, and PK were significantly higher in *C. gigas angulata* than in *C. gigas gigas* during acute heat stress. Moreover, these enzymes displayed high activities at 6 hr and decreased activities at 24 hr in both two subspecies (*p *<* *.01).

### 3.4 Expression of candidate genes

#### 3.4.1 Basal expression level

**Figure 4:**
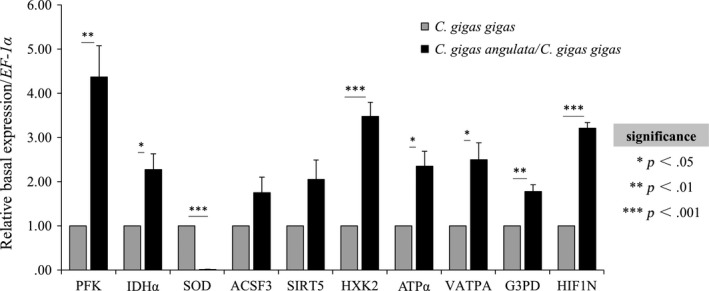
Basal expression levels of selected genes in *Crassostrea gigas gigas* and *C. gigas angulata* (*n* = 15). Asterisks indicate significant differences between these two subspecies (two‐tailed *t* test). Vertical bars represent standard errors. PFK, 6‐phosphofructokinase; HXK2, hexokinase type 2; VATPA, V‐type proton ATPase catalytic subunit A; SOD, superoxide dismutase; ACSF3, Acyl‐CoA synthetase family member 3; IDHα: isocitrate dehydrogenase [NAD] subunit alpha; SIRT5, NAD‐dependent protein deacetylase sirtuin‐5; ATPα, ATP synthase subunit alpha; G3PD, glyceraldehyde‐3‐phosphate dehydrogenase; HIF1N, hypoxia‐inducible factor 1‐ alpha inhibitor

Of the 10 genes investigated in this study, *C. gigas angulata* exhibited significantly higher basal expression levels of most candidate genes in comparison with *C. gigas gigas* (*PFK*,* IDH*α, *HXK2*,* ATP*α, *VATPA*,* G3PD*, and *HIF1N*, 1.78–4.37‐fold change; *p *<* *.05, Figure 4), while the expression of *SOD* in *C. gigas gigas* was 79.51‐fold higher than in *C. gigas angulata* (*p *<* *.001).

#### 3.4.2 Heat‐induced expression level

**Figure 5:**
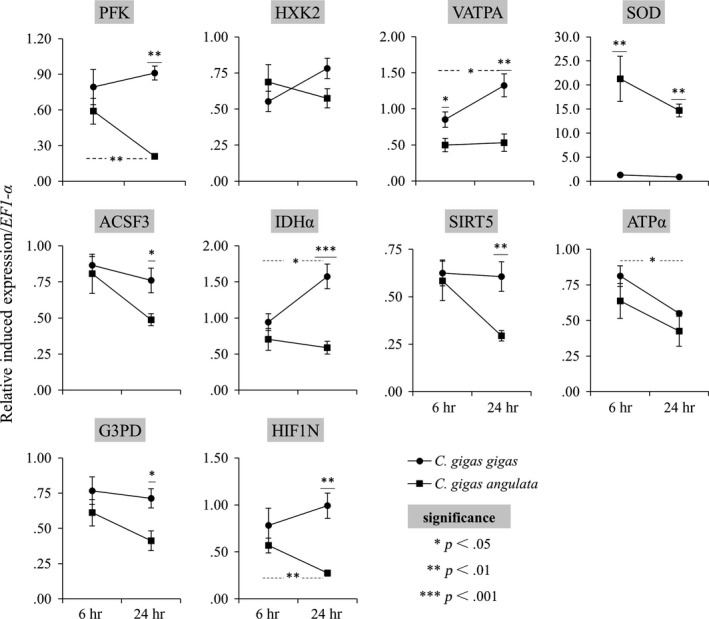
Induced expression of selected genes at 6 hr and 24 hr in response to acute heat stress in *Crassostrea gigas gigas* and *C. gigas angulata* (*n* = 15). Heat stress = 35°C. Asterisks indicate significant differences between subspecies (*p* < .05, ANOVA). Vertical bars represent standard errors. Abbreviations are same as above

There were obvious differences in the expression patterns between *C. gigas gigas* and *C. gigas angulata* (Figure 5). Almost all candidate genes exhibited a high degree of downregulation in *C. gigas angulata* in comparison with *C. gigas gigas* in response to thermal stress, with the exception that *C. gigas angulata* exhibited more than a 10‐fold higher induction in the expression of *SOD* mRNA (*p *<* *.01). Moreover, except for *HXK2* and *ATP*α mRNA, there were significant differences in the induced expression of the candidate genes between these two subspecies at both sampling times (*p *<* *.01). In addition, while the mRNA expression of nine genes (*PFK*,* HXK2*,* SOD*,* ACSF3*,* IDH*α, *SIRT5*,* ATP*α, *G3PD*, and *HIFIN*) was decreased to varying degrees in *C. gigas angulata* as the duration of heat stress increased, half of these genes (*PFK*,* HXK2*,* VATPA*,* IDH*α, and *HIFIN*) were upregulated in *C. gigas gigas*, the expression of *ATP*α was significantly down‐regulated, and the expression of the remaining genes did not change over time.

### 3.5 Genotype frequency of candidate genes

**Figure 6:**
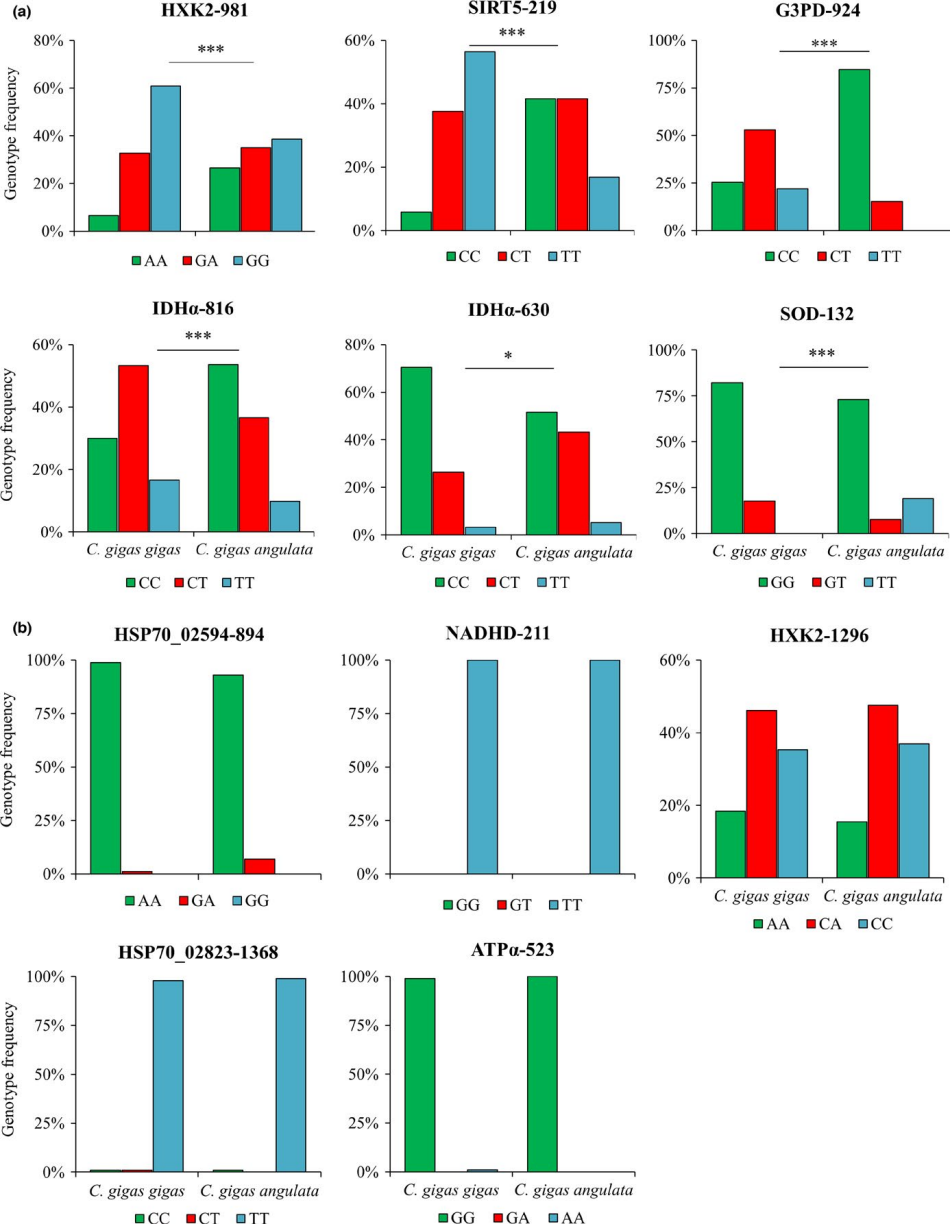
Genotype frequency of 11 single nucleotide polymorphisms (SNPs) of nine genes in *Crassostrea gigas gigas* and *C. gigas angulata*. Asterisks indicate significantly differentiated SNPs in (a), while no differences between these two subspecies are shown in (b). Pearson chi‐square test was used to determine difference of genotype frequency between *C. gigas gigas* and *C. gigas angulata* (*n* = 100)

**Figure 7:**
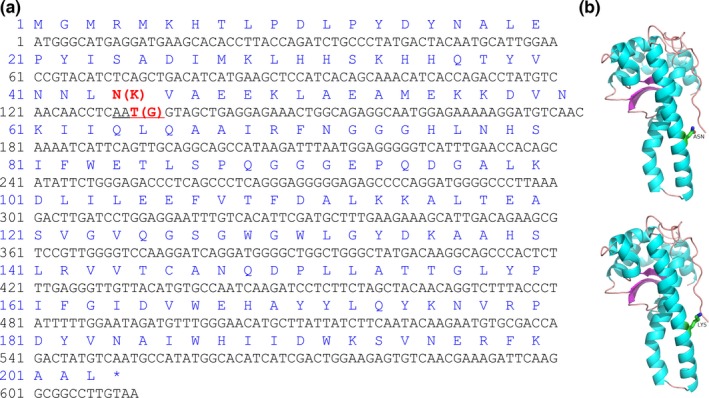
Nonsynonymous variations in DNA and amino acid sequences of gene *SOD* (a), and conformation variation of monomer protein of SOD (b)

Of the 203 SNPs in 22 genes with putatively different genotype frequencies between these two subspecies were identified (Table S2), while only 11 SNPs in nine candidate genes were successfully designed as primers (having passed the PAGE screening) and genotyped by HRM (Table S3). The genotype frequencies of six SNPs in five genes (*HXK2*‐981, *SOD*‐132, *G3PD*‐924, *IDH*α‐816, *IDH*α‐630, and *SIRT5*‐219) were significantly different between these two subspecies (Figure 6a, *p* < .01), whereas no differences were detected between these two subspecies in other five SNPs in five genes (*HSP70_02823*‐1368, *HSP70_02594*‐894, *HXK2*‐1296, *ATP*α‐523, and *NADHD*‐211) (Figure 6b). Five synonymous SNPs (Figure [Link], [Link], [Link], [Link]) and a nonsynonymous SNP (*SOD*‐132, Figure 7a) were detected with the absence of genotype TT in *C. gigas gigas* and resulted in 44th amino acid residues substitution between acidic asparagine and alkaline lysine that located in the α‐helix (Figure 7b).

## 4 DISCUSSION

Understanding the evolutionary potential of stressed species is of vital importance for the conservation of marine resources in face of global climate change and destructive human activities. Comprehensive studies in intra‐/interspecies variations from genotype to phenotype can provide mechanistic explanations for their distribution patterns (Bozinovic et al., 2011; Doney, 2010; Sanford & Kelly, 2011; Somero, 2012). Intraspecific physiological differences have been demonstrated to persist over the whole lifetime of an organism (Burton, Killen, Armstrong, & Metcalfe, 2011; White, Schimpf, & Cassey, 2013). Here, we further demonstrated that physiological and molecular divergence persisted even after one generation of common garden in identical conditions in two oyster subspecies. Also, we attempted to reveal the adaptive mechanisms of the thermal response in terms of energy metabolism in two oyster subspecies. The moderate degree of phenotypic plasticity observed in *C. gigas angulata* indicates it has higher adaptive potential in future coastal oceans (Careau et al., 2008; Schulte, 2015).

### 4.1 Tradeoffs in energy metabolism

For two oyster subspecies, increased respiration rates indicate that elevated temperature exerts strong effects on the metabolic rate, which support previous studies (Mao, Zhou, Yang, & Wang, 2006). Besides, all enzymes’ activities exhibited first rising and then decreasing pattern over the course of heat stress, indicating the transition from aerobic to anaerobic metabolism (Anestis et al., 2010; Sokolova & Pörtner, 2001; Sussarellu et al., 2012). Identical shifts were also documented by analyzing the expression patterns of *PK* mRNA and other heat responsive genes (Li, unpublished). Lastly, all genes involved in energy metabolism were downregulated in response to heat stress in both subspecies in comparison with basal levels, with the exception of the slightly elevated expression of *IDH*α at 24 hr in *C. gigas gigas*, thus supporting the notion that aerobic metabolism decreases in response to high‐temperature stress in marine animals (Tomanek, 2014). These findings provide direct evidence for the elucidation of energy‐limiting stress tolerance in the oyster, where high temperature stress induces tradeoffs between aerobic and anaerobic energy pathways. Furthermore, divergent expression patterns between energy metabolic genes and oxidative stress gene were observed in both subspecies, which further demonstrating tradeoffs between energy metabolism and protective responses under elevated temperature in the oyster.

However, the total protein and MDA concentrations did not appear to be temperature sensitive and did not show divergence between two oyster subspecies under acute heat stress. The low resolution of MDA was also observed in hypoxia‐treated oysters (Sussarellu et al., 2012), although it has been proven to be an effective marker of oxidative stress in the mud clam (Abele, Heise, Pörtner, & Puntarulo, 2002).

### 4.2 Adaptive phenotypic plasticity in energy metabolism

In this study, two oyster subspecies exhibited different extents of energetic/metabolic regulation. The southern oysters, *C. gigas angulata*, exhibited a higher respiration rate than its northern counterpart, *C. gigas gigas*, both in ambient and high temperature. In line with our data, a temperate population of amphipods also exhibited higher metabolic rates in comparison with congeneric subarctic populations (Rastrick & Whiteley, 2011). Similarly, southern oysters exhibited higher activities of all three enzymes (ATPase, PK, and SOD) in comparison with northern oysters. Identical proteomic evidence was detected in the blue mussel, where lower levels of key metabolic enzymes involved in energy production were observed in the cold‐adapted *Mytilus galloprovincialis* in response to increased temperature in comparison with its southern counterpart, *M. trossulus* (Tomanek & Zuzow, 2010). Moreover, differential expression patterns of key energy metabolic genes were observed in these two subspecies. Northern oysters exhibited lower basal levels of expression, and a lower degree of downregulation in comparison with southern counterpart, who exhibited higher basal levels of expression and a higher extent of downregulation for thermally induced expression of these genes. Higher constitutive expression of genes involved in energy metabolism in warmer congeners was also found in corals inhabiting warmer inshore locations (Kenkel et al., 2013). However, oxidative stress gene *SOD* displayed distinct expression patterns to these genes, where northern oysters showed higher basal levels of expression and a lower degree of induced expression, while southern oysters exhibited lower basal levels and a higher degree of induced expression. All above differences could be best explained as a protective mechanism, as higher metabolic rate, enzymes’ activities, upregulation of induced expression in oxidative gene and downregulation of induced expression in energy metabolic genes in organisms inhabiting extremely high‐temperature habitats could alleviate potential oxidative damage from elevated amounts of ROS (Abele et al., 2002). Also, low basal expression levels of key metabolic genes in the northern oyster suggest that it tends to conserve energy reserves during ambient conditions, while a contrasting expression pattern observed in the southern oyster, indicating the increased energy gain from the habitats (Marshall & McQuaid, 2011). Evolutionary divergences in these differences could also be supported by the fact that the abundance and diversity of phytoplankton are greater in the southern sea in comparison with the northern sea of China (data downloaded from the State Oceanic Administration People's Republic of China).

Interestingly, southern oysters consistently exhibited higher flexibility in metabolic rate, enzymatic activities and induced expression levels of candidate genes (high extent of up‐ or downregulation) in response to elevated temperatures than northern oysters, indicating this subspecies has developed a higher degree of phenotypic plasticity (Careau et al., 2008), thereby supporting the conclusions of the accompanying paper (Li, unpublished). Importance of adaptive plasticity in prompting diversification and speciation at different levels of biological organization has garnered widespread acceptance (Pfennig et al., 2010). High levels of plasticity place population close to an adaptive peak and increase the probability of population persistence but reduce the likelihood of genetic change. While organisms that express moderate levels of plasticity are optimal in permitting population survival in encounter future climate change (Price, Qvarnstrom, & Irwin, 2003). We attempt to reveal adaptive mechanisms underline divergence between two oyster subspecies in the view of energy metabolism and show that *C. gigas angulata* has higher adaptive potential than its northern counterpart in face of global warming (Dayan, Crawford, & Oleksiak, 2015; Evans & Hofmann, 2012; Healy & Schulte, 2015).

### 4.3 Potential adaptive mechanisms in sequence variations

Considerable quantities of SNPs detected in candidate genes provide further evidence that the oyster possesses a high degree of sequence variation (Wang et al., 2015). We identified obvious divergences in the genotype frequencies of six SNPs in five energetic and oxidative stress genes of two oyster subspecies and further considerably validated in the timescale. It is not likely to be synthetic sequence variations due to they were identified by two independent wild oyster (Bozinovic et al., 2011; Sanford & Kelly, 2011). Similar SNPs in genes involved in key energy metabolism were documented in intertidal populations of the acorn barnacle (*Semibalanus balanoides*) (Schmidt & Rand, 2001), and broadly distributed mussel (*Mytilus edulis*) (Hilbish & Koehn, 1985) and Atlantic herring (*Clupea harengus*) (Limborg et al., 2012). These heritable genomic variations suggest *C. gigas angulata* may evolve a moderate level of plasticity (Price et al., 2003).

The amino acid changes induced by nonsynonymous variation in *SOD‐132* lead to protein conformation changes, which possibly influence the enzymatic function and activity. Synonymous SNPs were detected in four energy metabolic genes, and they may also regulate molecular functions and even produce phenotypic variations through changing the ability of RNA binding proteins to recognize the transcript and further alter the stability of transcripts (Chen, Davydov, Sirota, & Butte, 2010; Hunt, Sauna, Ambudkar, Gottesman, & Kimchi‐Sarfaty, 2009). Alternatively, these synonymous variations in functionally conserved genes can potentially interpret observed adaptive phenotypic plasticity in this study. However, functional validations are demanded in the future studies. Besides, no SNPs in *HSPs* were validated in the oyster in the present study, while being found in the Atlantic herring (Limborg et al., 2012). It is possible that, in general, the oyster expands *HSP* gene families as an alternative to changes in genomic structure (Guo et al., 2015; Zhang et al., 2012). To deeply dissect the role of temperature in the relationship between the plasticity of energy metabolism and adaptive divergence in the oyster, additional investigations are required (e.g., other potentially indicative adaptive markers, validating SNPs’ function in candidate genes, et al.). Besides, more generations of common‐garden rearing should be conducted due to limited capacity of negating potential plastic maternal effects (Sanford & Kelly, 2011).

## 5 CONCLUSION

In summary, we explored the adaptive divergence, from the view of energy metabolism, of two oyster subspecies (*C. gigas gigas* and *C. gigas angulata*) that are broadly distributed along the northern and southern coastal areas of China, respectively. All data indicate that southern oysters have evolved a moderate degree of phenotypic plasticity, which has higher adaptive potential in future coastal oceans. In addition, metabolic tradeoffs were detected in response to heat stress. Moreover, identification and validation of synonymous and nonsynonymous sequence variations in these genes help understand their adaptive mechanisms. However, studies focusing on functional validations are required. Our integrative study further elucidates the importance of adaptive plasticity in evolutionary potential and provides fundamental knowledge regarding resource management of costal invertebrates under the scenario of global warming.

## DATA ACCESSIBILITY

Detailed information regarding the 203 SNPs, with differing genotype frequencies between *C. gigas gigas* and *C. gigas angulata* in 22 genes, has been provided as online supplemental data.

## CONFLICT OF INTEREST

The authors declare that they have no conflict of interests.

## AUTHOR CONTRIBUTIONS

Li Li and Guofan Zhang designed the study; Ao Li carried out the laboratory work and performed data analysis; Ao Li, Li Li, and Guofan Zhang wrote and revised the manuscript; Kai Song called the SNPs; Li Li and Wei Wang collected the oysters and oversaw the spawning. All authors gave the final approval for publication.
