# Supplementary figures and images for: Temperature, energy metabolism, and adaptive divergence in two oyster subspecies
Source: Ecol Evol. 2017 Jun 30;7(16):6151–62. doi: 10.1002/ece3.3085 (PMC5574764; doi:10.1002/ece3.3085)

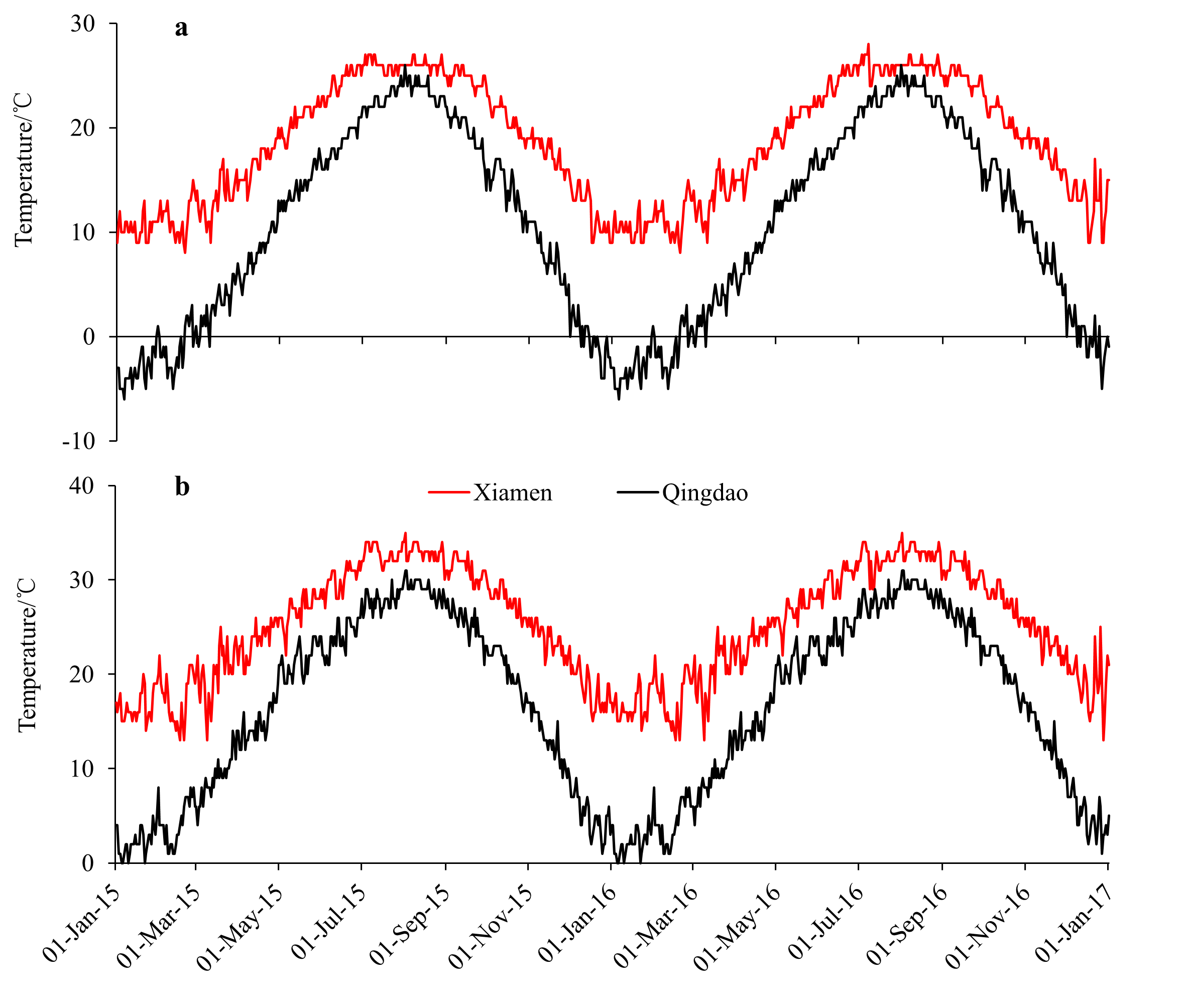

Supplement: Supplementary file 1 [file ECE3-7-6151-s001.tif]

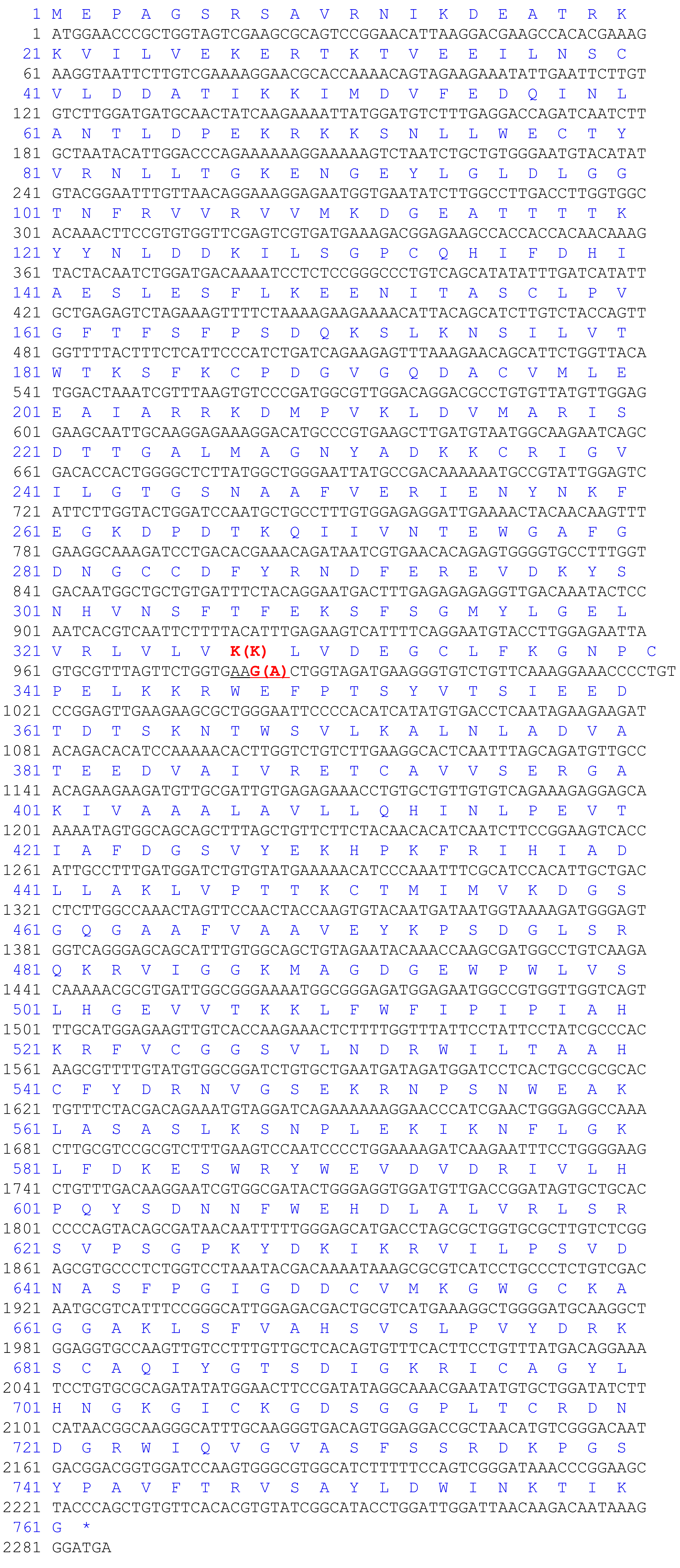

Supplement: Supplementary file 2 [file ECE3-7-6151-s002.tif]

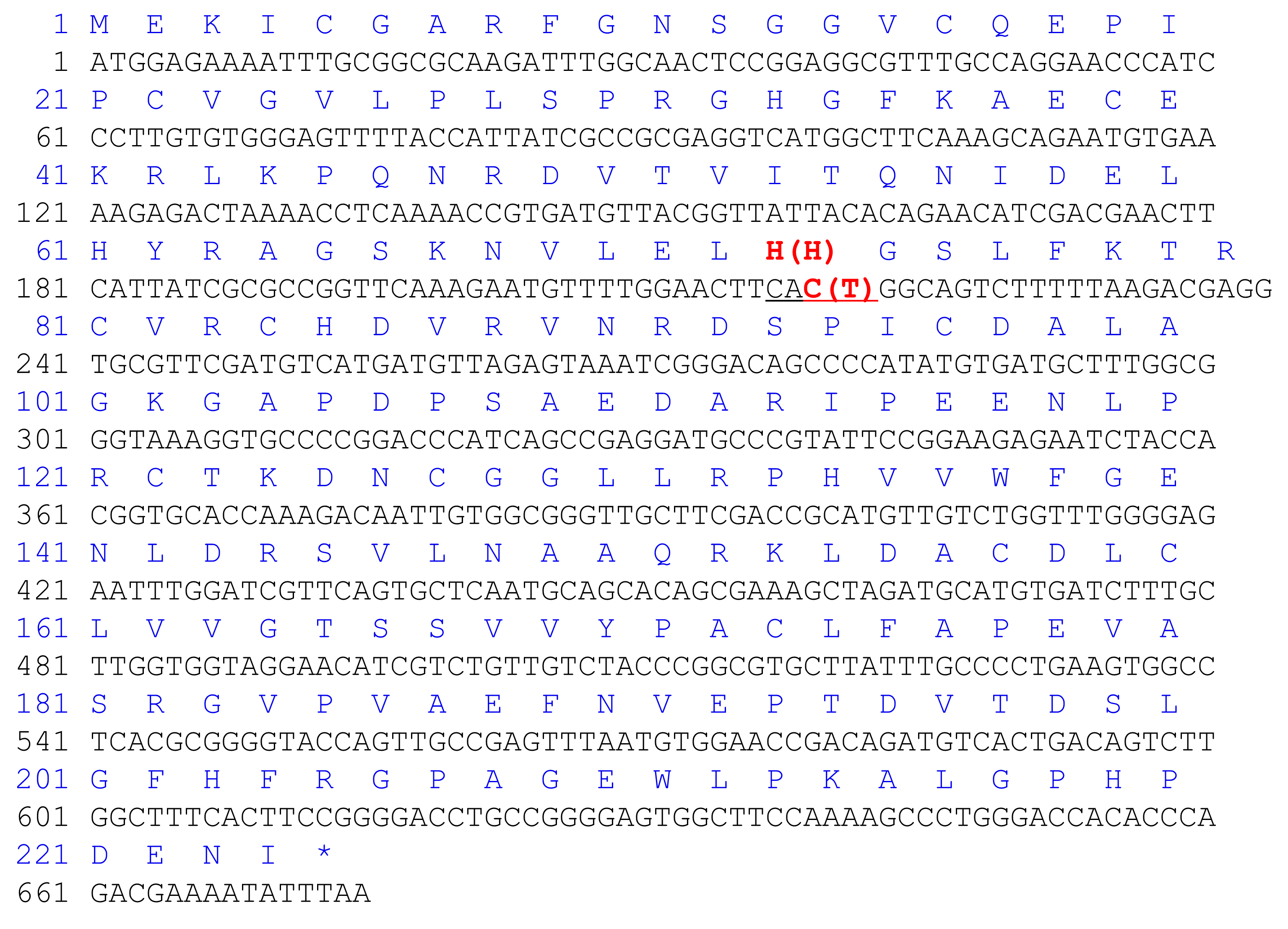

Supplement: Supplementary file 3 [file ECE3-7-6151-s003.tif]

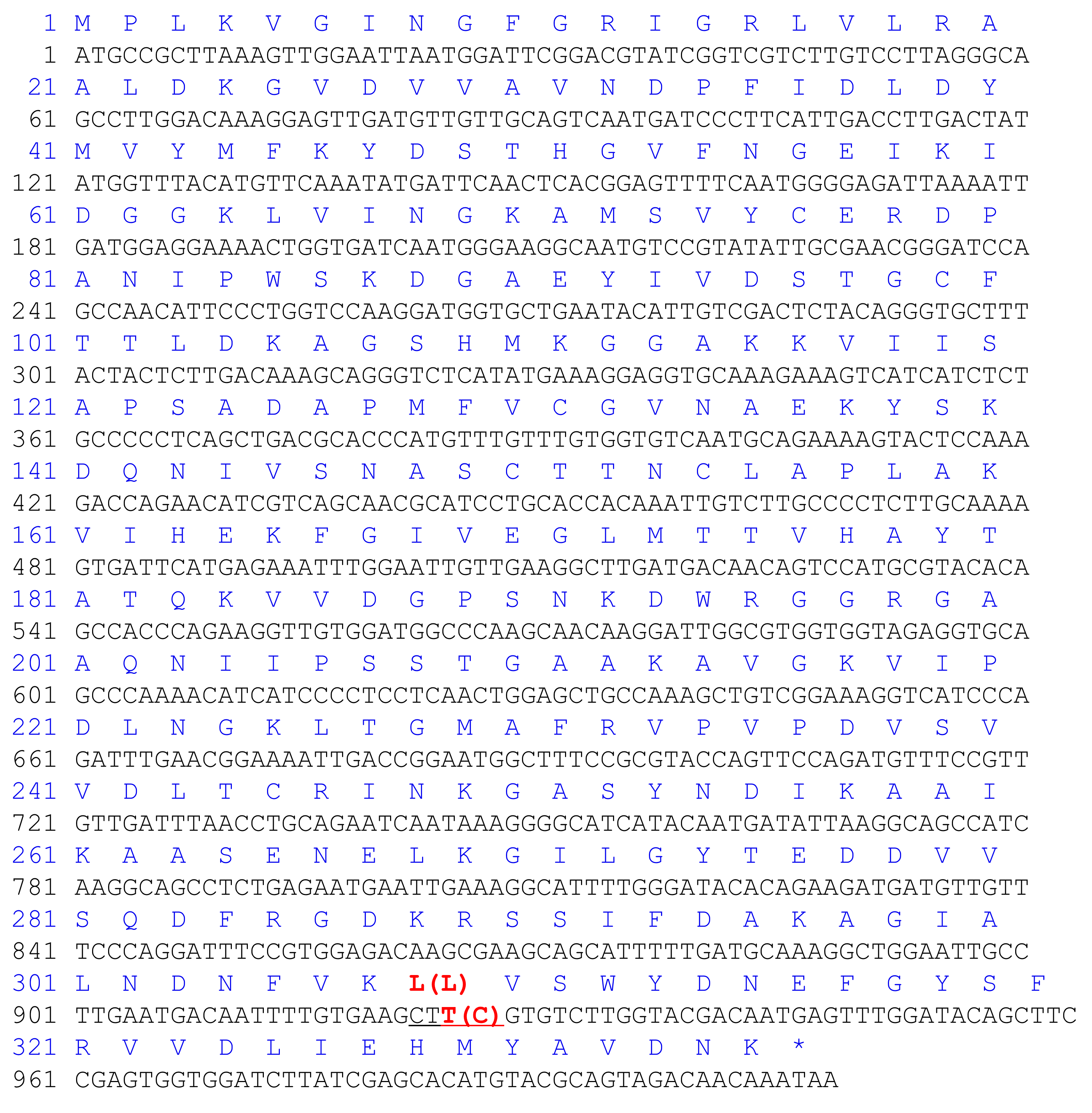

Supplement: Supplementary file 4 [file ECE3-7-6151-s004.tif]

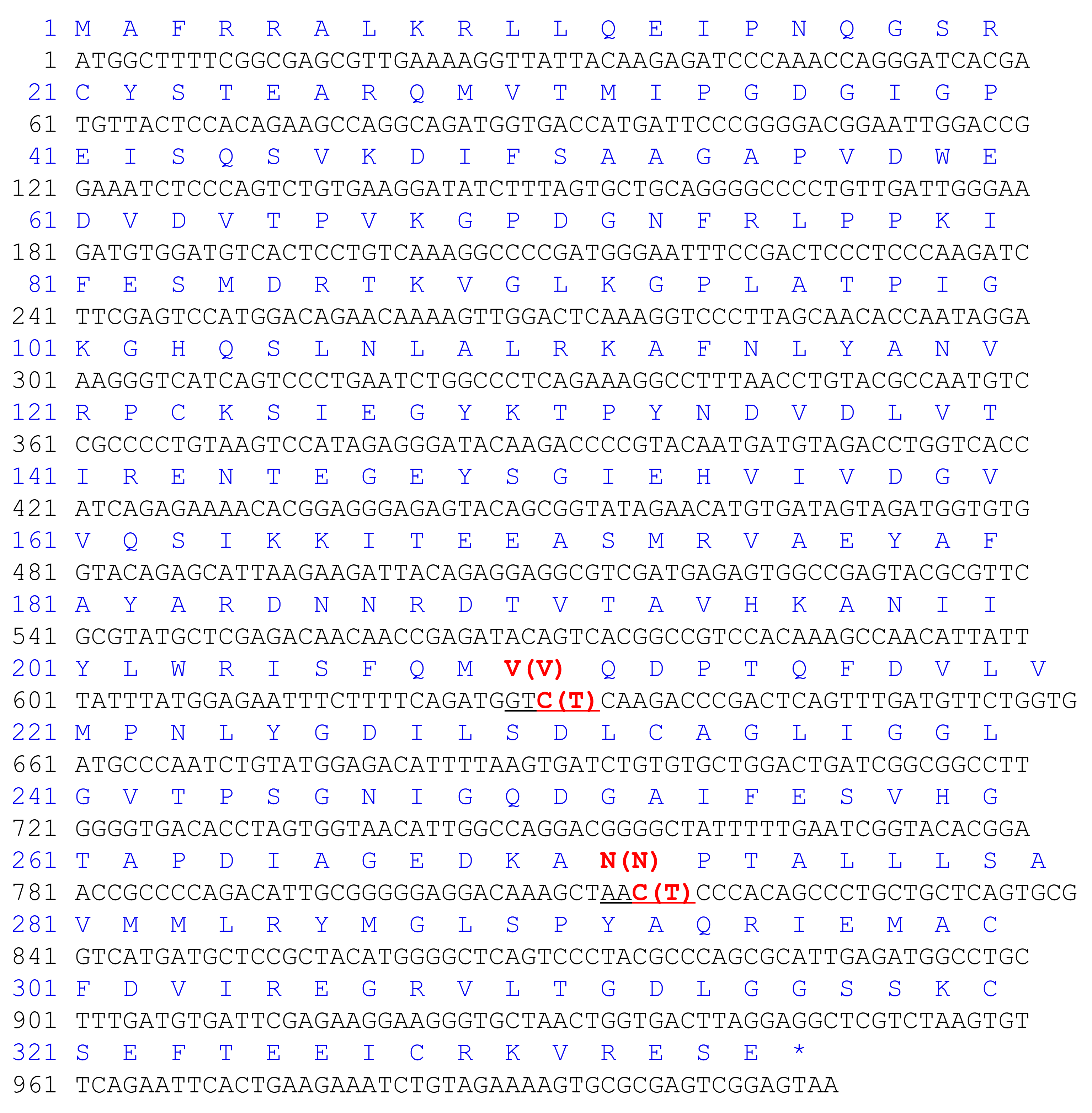

Supplement: Supplementary file 5 [file ECE3-7-6151-s005.tif]
